# Anti-Inflammatory Effect of Melittin on *Porphyromonas Gingivalis* LPS-Stimulated Human Keratinocytes

**DOI:** 10.3390/molecules23020332

**Published:** 2018-02-05

**Authors:** Woon-Hae Kim, Hyun-Jin An, Jung-Yeon Kim, Mi-Gyeong Gwon, Hyemin Gu, Minji Jeon, Min-Kyung Kim, Sang-Mi Han, Kwan-Kyu Park

**Affiliations:** 1 Department of Pathology, College of Medicine, Catholic University of Daegu, Daegu 42472, Korea; kimwoonhae@cu.ac.kr (W.-H.K.); ahj119@cu.ac.kr (H.-J.A.); jy1118@cu.ac.kr (J.-Y.K.); daldy99@naver.com (M.-G.G.); asdf8760@naver.com (H.G.); minzy1081@cu.ac.kr (M.J.); 2 Department of Pathology, College of Medicine, Dongguk University, Gyeongju-si 38066, Korea; minkyungk76@naver.com; 3 Department of Agricultural Biology, National Academy of Agricultural Science, RDA, 300, Nongsaengmyeong-ro, Wansan-gu, Jeonju-si, Jeollabuk-do 55365, Korea; sangmih@korea.kr

**Keywords:** keratinocytes, lipopolysaccharides, melittin, periodontitis, *Porphyromonas gingivalis*

## Abstract

Periodontitis is a chronic inflammatory disease that contributes to the destruction of the gingiva. *Porphyromonas gingivalis* (*P. gingivalis*) can cause periodontitis via its pathogenic lipopolysaccharides (LPS). Melittin, a major component of bee venom, is known to have anti-inflammatory and antibacterial effects. However, the role of melittin in the inflammatory response has not been elucidated in periodontitis-like human keratinocytes. Therefore, we investigated the anti-inflammatory effects of melittin on a *P. gingivalis* LPS (PgLPS)-treated HaCaT human keratinocyte cell line. The cytotoxicity of melittin was measured using a human keratinocyte cell line, HaCaT, and a Cell Counting Kit-8. The effect of melittin on PgLPS-induced inflammation was determined with Western blot, real-time quantitative PCT, and immunofluorescence. PgLPS increased the expression of toll-like receptor (TLR) 4 and proinflammatory cytokines, such as tumor necrosis factor-α (TNF-α), interleukin (IL)-6, IL-8, and interferon-γ (IFN-γ). Moreover, PgLPS induced activation of the nuclear factor kappa-light-chain-enhancer of activated B cells (NF-κB), extracellular signal-regulated kinase (ERK), and protein kinase B/Akt. Melittin also inhibited the expression of proinflammatory cytokines by suppressing the activation of the NF-κB signaling pathway, ERK, and Akt. Melittin attenuates the PgLPS-induced inflammatory response and could therefore be applied in the treatment of periodontitis for anti-inflammatory effects.

## 1. Introduction

Periodontitis is a chronic inflammatory disease caused by a multifactorial process involving interactions between oral keratinocytes and the microorganisms that colonize the mouth [1,2]. *Porphyromonas gingivalis* (*P. gingivalis*) invades periodontal tissues and releases toxic substances, such as lipopolysaccharide (LPS) , leading to periodontal inflammation and alveolar bone destruction [3]. Oral keratinocytes are on the frontline of the immune defense system against bacterial infections via toll-like receptors (TLRs), by producing a variety of pro-inflammatory cytokines and chemokines [4]. In gingival epithelial cell lines, *P. gingivalis* LPS (PgLPS) upregulates interleukin (IL)-6, IL-8, tumor necrosis factor (TNF)-α, and interferon (IFN)-γ gene expression and protein synthesis [5,6]. In addition, these cytokines activate neutrophils and macrophages to enhance the inflammatory response [7].

The pathogenesis of periodontitis is associated with increased levels of inflammatory cytokines and their destructive response in gingival tissues [8]. Conventional treatment for periodontal disease includes dental scaling of the subgingival tooth to eliminate the dental plaque biofilm, or surgical procedures in cases of severe loss of tooth-supporting tissue [9]. Despite these clinical interventions, periodontitis is often uncontrolled or recurrent [10,11]. Gingival tissues in patients with periodontitis show greater increases in pro-inflammatory cytokines, such as IL-1, IL-6, IL-8, and TNF-α, as well as other inflammatory mediators, compared to gingival tissues in healthy individuals [12]. Thus, numerous studies have used animal models to investigate anti-inflammatory therapies for periodontitis [13,14,15]. There are no definitive anti-inflammatory agents for this condition; however, bee venom and its major component, melittin, have recently emerged as antibacterial and anti-inflammatory agents.

Melittin is the major component (50% of dry weight) of bee venom [16]. Bee venom is a natural toxin produced by the honeybee (*Apis mellifera*), and has been widely used in Eastern medicine [17]. Researchers have demonstrated that bee venom and melittin have pharmacological effects on various diseases [18,19]. Melittin induces cell-cycle arrest and apoptosis in various cancer cells [20,21], and has therapeutic effects in mouse models of liver cirrhosis [22], atherosclerosis [23], and *Propionibacterium acnes*-induced inflammation [24]. Although bee venom has anti-inflammatory effects on PgLPS-treated human keratinocytes [25], no attempt has yet been made to demonstrate that melittin is an effective anti-inflammatory agent for *P. gingivalis*-induced periodontitis. Therefore, this study aimed to evaluate the effect of melittin on the expression of TLR-4 and inflammatory cytokines in PgLPS-treated human keratinocytes.

## 2. Results

### 2.1. Effect of Melittin on HaCaT Cell Viability

**Figure 1:**
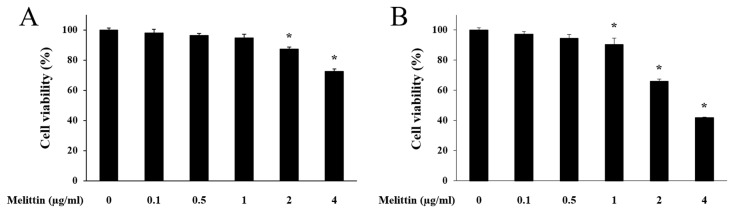
Cytotoxicity of melittin in HaCaT cells. HaCaT cells were treated with different concentrations of melittin for 8 h (**A**) and 24 h (**B**). Results are expressed as the mean ± SEM of three independent determinations. * *p* < 0.05 compared to the untreated group.

When melittin treatment was performed for 8 h, HaCaT cell viability was decreased at 2 μg/mL and 4 μg/mL melittin concentrations. However, no significant viability changes were detected at melittin concentrations below 2 μg/mL (Figure 1A). When melittin treatment was performed for 24 h, HaCaT cell viability was reduced at the 1 μg/mL, 2 μg/mL, and 4 μg/mL concentrations (Figure 1B). Thus, the following experiments were performed with doses of <1 μg/mL of melittin, for 8 h each.

### 2.2. Melittin Inhibits PgLPS-Induced Expression of TLR-4 and Inflammatory Cytokines

**Figure 2:**
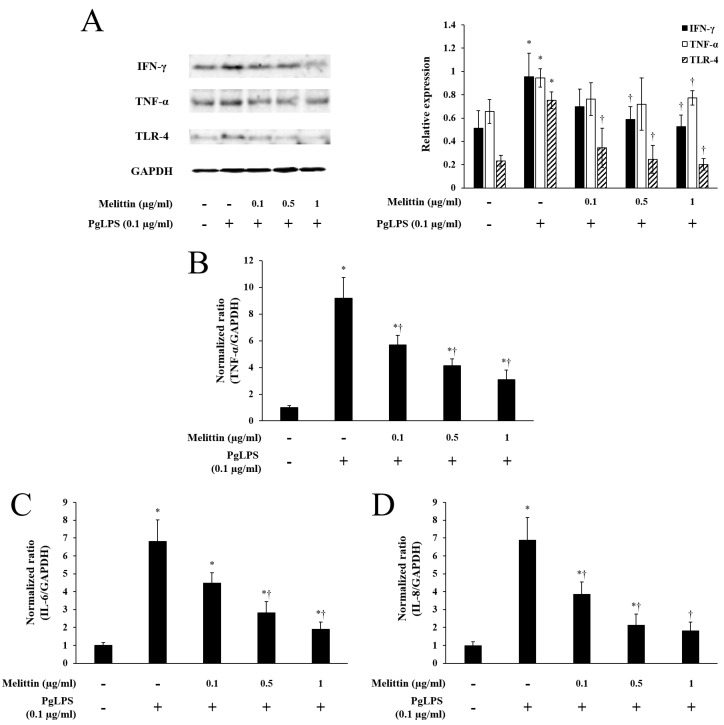
Effects of melittin on *Porphyromonas gingivalis* lipopolysaccharide (PgLPS)-induced expression of toll-like receptor (TLR)-4 and inflammatory cytokines. (**A**) Representative Western blot images show the effects of PgLPS and melittin on the protein expression of TLR-4, interferon (IFN)-γ, and tumor necrosis factor (TNF)-α. The bar graph shows quantitative signal intensities of the proteins after normalization with GAPDH, respectively. (**B–D**) Quantitative real-time PCR was used to determine the effects of PgLPS and melittin on mRNA expression of TNF-α, IL-6, and IL-8. The graphs summarize the analysis of relative TNF-α, IL-6, and IL-8 mRNA expression, normalized to GAPDH, respectively. −: untreated, +: treated. Results are expressed as the mean ± SEM of three independent determinations. * *p* < 0.05 compared to the untreated group. † *p* < 0.05 compared to the PgLPS group.

Using a Western blot analysis, the PgLPS-treated group showed increased protein expression of IFN-γ, TNF-α, and TLR-4 compared to the untreated group. However, melittin decreased the expression of these proteins (Figure 2A). Quantitative real-time PCR showed that PgLPS induced the RNA expression of TNF-α, IL-6, and IL-8, compared to the PgLPS-untreated group (Figure 2B–D). However, melittin significantly inhibited RNA expression of TNF-α and IL-8 in a dose-dependent manner (Figure 2B,D). Melittin reduced the RNA expression of IL-6, statistically significantly at 0.5 μg/mL and 1 μg/mL concentrations (Figure 2C).

### 2.3. Melittin Inhibits PgLPS-Induced Activation of the NF-κB Signaling Pathway, Akt, and ERK

**Figure 3:**
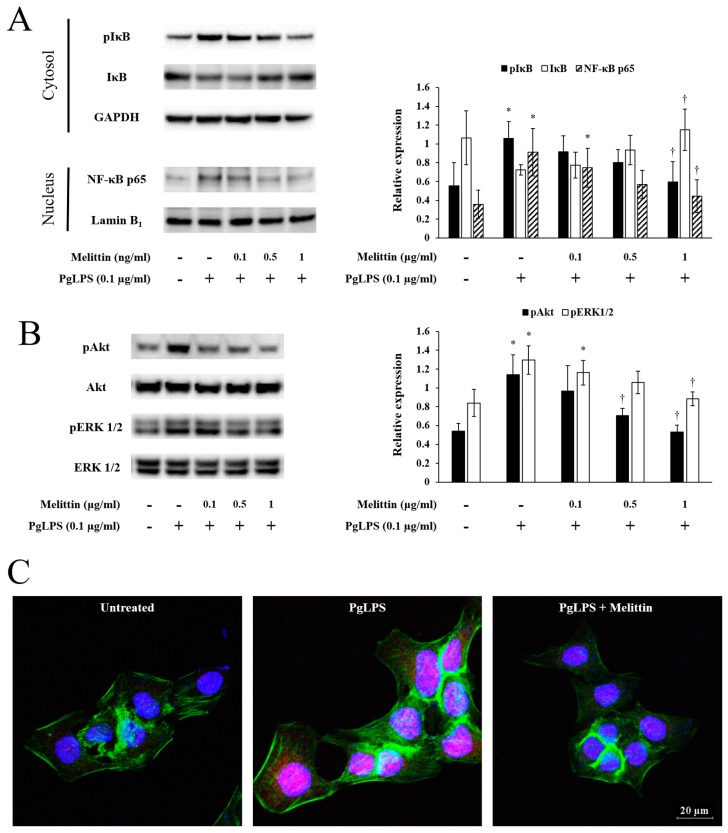
Effects of melittin on PgLPS-induced activation of NF-κB signaling pathway, Akt, and ERK1/2. Representative Western blot images show the effects of PgLPS and melittin on the activation of cytosolic NF-κB inhibitor (IκB), nuclear NF-κB (**A**); Akt, and ERK1/2 (**B**). The bar graphs show quantitative signal intensities of the proteins after normalization with glyceraldehyde 3-phosphate dehydrogenase (GAPDH), Lamin B_1_, Akt, and ERK 1/2, respectively. -: untreated, +: treated. * *p* < 0.05 compared to the untreated group. † *p* < 0.05 compared to the PgLPS group. (**C**) Representative immunofluorescence images show the effects of PgLPS and melittin on the activation of NF-κB (labeled with Alexa Fluor 647, red) in HaCaT cells. The nuclei were labeled with Hoechst 33342 (blue). β-actin was labeled with Alexa Fluor 488 (green). PgLPS: 0.1 μg/mL of *P. gingivalis* lipopolysaccharides, Melittin: 1 μg/mL of melittin.

PgLPS increased the expression of phosphorylated (p) NF-κB inhibitor (IκB) in the cytoplasm, while PgLPS-induced pIκB expression was decreased by melittin. The expression pattern of IκB proteins was opposite that of pIκB. PgLPS increased NF-κB proteins in the nucleus, compared with the PgLPS-untreated group. However, melittin inhibited the PgLPS-induced expression of NF-κB proteins (Figure 3A) as well as pAkt and pERK1/2 proteins (Figure 3B). In the immunofluorescence analysis, PgLPS increased the expression of NF-κB proteins in the nucleus, while PgLPS-induced NF-κB protein expression was decreased by the 1 μg/mL melittin concentration (Figure 3C).

## 3. Discussion

This study evaluated the effects of melittin on PgLPS-induced inflammation in human keratinocytes. PgLPS induced the expression of TLR-4 and inflammatory cytokines through the activation of the NF-κB signaling pathway, Akt, and ERK1/2. However, melittin inhibited PgLPS-induced expression of TLR-4 and inflammatory cytokines by blocking the NF-κB signaling pathway, Akt, and ERK1/2.

Gingival tissue mainly consists of gingival epithelial cells, including keratinocytes, which directly interact with *P. gingivalis* and its virulence factors, such as LPS, in periodontal tissues [26,27]. PgLPS is a crucial factor in the development of periodontitis, and has been reported to promote the production of IL-1, IL-6, and IL-8 [28]. Roberts et al. [29] showed that pro-inflammatory cytokines, including IL-1α, IL-1β, IL-6, IL-8, IL-12, IL-13, TNF-α, and IFN-γ, were increased in chronic periodontitis. Consistent with previous findings, the present study showed that PgLPS increased the expression of IL-6, IL-8, TNF-α, and IFN-γ in human keratinocytes. TLRs are key proteins involved in the host defense mechanism against various pathogens, including bacteria [30]. TLR-2 is activated mostly by pathogen-associated substrates from Gram-positive bacteria, while TLR-4 is activated by enterobacterial LPS activation [31], as well as PgLPS [32]. Thus, in the present study, PgLPS was used to activate TLR-4 and to promote inflammation in keratinocytes.

During the inflammatory response, IFN-γ plays an important role in the host defense mechanism against bacteria and their virulence factors by activating phagocytes and the inflammatory reaction [33]. Bacteria and their virulence factors induce the pro-inflammatory cytokines, IL-8 and TNF-α, which may play a role in the chemoattraction and maturation of inflammatory cells [34]. In addition, previous studies have demonstrated how LPS and bacteria directly contribute to the production of TNF-α, IL-1β, IL-8, and IFN-γ via TLR expression [35,36], suggesting that inhibiting TLR can help control inflammatory mediators [37,38].

Activation of TLRs usually activates the NF-κB, Akt, and MAPK ERK1/2 signaling pathways, leading to the upregulation of inflammatory gene expression, which is essential for the innate immune response to inflammation [39]. NF-κB comprises a family of inducible transcription factors that serve as important regulators in the host immune and inflammatory responses [40]. IκB is a member of the NF-κB signaling pathway, and functions as an inhibitor of NF-κB by masking the nuclear localization signals of NF-κB proteins and keeping them sequestered in an inactive state in the cytoplasm [41]. When IκB is phosphorylated by IκB kinase, NF-κB is dissociated from IκB and migrates into the nucleus [42]. Thus, reduced expression of nuclear NF-κB and cytoplasmic IκB can be an effective strategy for inhibition of inflammation.

Besides NF-κB, TLRs also activate Akt and MAPK ERK1/2, which are involved in cellular events, such as proliferation, survival, differentiation, and inflammation [43,44]. Activation of Akt and ERK1/2 increases the production of pro-inflammatory mediators, such as TNF-α and IL-1β [45,46]. The modulation of these pathways provides a potential therapeutic approach for the treatment of inflammatory disease [47]. In the present study, the expression of Akt and ERK1/2 was increased by PgLPS, but decreased by melittin.

Despite the results of our experiments showing that melittin has anti-inflammatory effects in a dose-dependent manner, there is a point that should not be overlooked. Based on our experimental results, the therapeutic window of melittin is so narrow that finding the optimal dose for clinical use or an in vivo test may be difficult. Therefore, it is necessary to be cautious when determining the concentration of melittin for clinical use or in vivo experiments. Of course, clinical use or in vivo and in vitro experiments may be different, but it is important that our study has shown that melitin has anti-inflammatory properties. This result may be enough evidence to study the therapeutic efficacy of melitin for PgLPS-induced inflammation in clinical use or in an in vivo experiment. 

In summary, *P. gingivalis* and its major virulence factor, PgLPS, crucially contribute to inflammation through increased expression of TLR-4 and inflammatory cytokines, including IL-6, IL-8, TNF-α, and IFN-γ. It has been demonstrated that melittin has an anti-inflammatory effect by inhibiting inflammatory cytokines. Moreover, the activation of inflammatory-cytokine-related signaling pathways, including NF-κB, Akt, and ERK1/2, was decreased by melittin in PgLPS-treated human keratinocytes. These results demonstrate that melittin inhibits inflammatory cytokines by blocking their prime signaling pathways, suggesting that melittin has anti-inflammatory effects on PgLPS-stimulated human keratinocytes. In addition, our results indicate that melittin can protect keratinocytes against PgLPS-mediated injury. Therefore, melittin can be regarded as an alternative anti-inflammatory agent for the treatment of periodontitis. Authors should discuss the results and how they can be interpreted in perspective of previous studies and of the working hypotheses. The findings and their implications should be discussed in the broadest context possible. Future research directions may also be highlighted.

## 4. Materials and Methods 

### 4.1. Cell Culture and Reagents

HaCaT cells (CLS, Eppelheim, Germany) were cultured in Dulbecco’s modified Eagle’s medium (DMEM; GE Healthcare Life Sciences HyClone Laboratories, South Logan, UT, USA) supplemented with 10% fetal bovine serum and 1% antibiotics at 37 °C in a humidified 5% CO_2_ incubator. The HaCaT cells were seeded at 1.0 × 10^6^ cells per 3 mL of complete medium in a 100-mm TC-treated cell-culture dish. The medium was changed 24 h later with serum-free medium containing the indicated concentrations of melittin (0.1, 0.5, and 1 μg/mL; Enzo Life Sciences, Farmingdale, NY, USA). After 1 h, the cells were co-treated with 100 ng/mL of PgLPS (InvivoGen, San Diego, CA, USA). After 7 h, the cells were collected for the next experiment.

### 4.2. Cell Viability Test

The cell viability of HaCaT was determined with a CCK-8 assay (Dojindo, Kumamoto, Japan). The cells were seeded in a 96-well plate at 5.0 × 103 cells/well and pre-incubated for 24 h. After pre-incubation, the cells were treated with melittin (0.1, 0.5, 1, and 2 μg/mL) and 100 ng/mL of PgLPS for 8 h or 24 h. After treatment, 10 μL of WST-8 solution [2-(2-methoxy-4-nitrophenyl)-3-(4-nitrophenyl)-5-(2,4-disulfophenyl)-2H-tetrazolium, monosodium salt] was added to each well, and the cells were incubated for an additional 4 h at 37 °C. The cell viability values were measured by absorbance at 450 nm using a microplate reader.

### 4.3. Quantitative Real-Time Polymerase Chain Reaction

Total mRNA was extracted from HaCaT cells with TRIzol reagent (Thermo Fisher Scientific, Waltham, MA, USA), according to the manufacturer’s recommendations. A reverse transcription reaction was performed with AccuPower RT Premix and Oligo dT18 (Bioneer, Daejeon, Korea), according to the manufacturer’s instructions. Real-time PCR was performed in a LightCycler Nano System (Roche Applied Science, Mannheim, Germany) with LightCycler DNA Master SYBR GREEN I (Roche Applied Science, Mannheim, Germany). The PCR mixtures contained 100 ng of cDNA and 0.5 μM each of the forward and reverse primers. The samples were denatured at 95 °C for 10 min, followed by 45 cycles of annealing and extension at 95 °C for 20 s, 60 °C for 20 s, and 72 °C for 20 s. Expression values were normalized to glyceraldehyde 3-phosphate dehydrogenase (GAPDH). Quantitative real-time PCR products were further confirmed by melting curve analysis. The analyzed target genes were TNF-α, forward, 5’-AGTGGTGCCAGCCGATGGGTTGT-3’, and reverse, 5’-GCTGAGTTGGTCCCCCTTCTCCAG-3’; IL-8, forward, 5’-TCCAATTCGGGAGACCTCTA-3’, and reverse, 5’-TAGGCATCACTGCCTGTCAA-3’; IL-6, forward, 5’-GGTACATCCTCGACGGCATCT-3, and reverse, 5’-GTGCCTCTTTGCTGCTTTCAC-3’; GAPDH, forward, 5’-GGAGCCAAAAGGGTCATCAT-3’, and reverse, 5’-GTGATGGCATGGACTGTGGT-3’.

### 4.4. Western Blot

Protein samples were prepared from the cultured HaCaT cells with a protein extraction buffer (Cell Lytic™ M; Sigma, St. Louis, MO, USA), in accordance with the instruction manual. The protein concentration of the samples was measured with a Bradford assay (Bio-Rad Laboratories, Hercules, CA, USA) using a spectrophotometer with optimal density at 595 nm. The protein samples were separated on precast gradient polyacrylamide gels (Bolt™ 4–12% Bis-Tris Plus Gels; Thermo Fisher Scientific, Waltham, MA, USA) and transferred to nitrocellulose membranes (GE Healthcare, Little Chalfont, Buckinghamshire, UK) by using the Bolt™ Mini Blot Module and Mini Gel Tank (Thermo Fisher Scientific, Waltham, MA, USA), in accordance with the manufacturer’s recommendations. The membrane was blocked in 5% bovine serum albumin (BSA; Sigma, St. Louis, MO, USA). The blocked membrane was probed with a primary antibody and a horseradish peroxidase-conjugated secondary antibody. Following a repeat of the washing step, the membrane was kept in enhanced chemiluminescence detection reagents (Thermo Fisher Scientific, Waltham, MA, USA) for 1 min. Signal intensity was measured with an image analyzer (ChemiDoc™ XRS+; Bio-Rad Laboratories, Hercules, CA, USA). The primary antibodies used in the present study were purchased from Abcam (anti-IFN-γ, ab9657; anti-TNF-α, ab1793; Cambridge, Cambridgeshire, UK), Cell Signaling Technology (anti-Akt, #9272; anti-phospho-Akt, #9271; anti-ERK, #4695; anti-phospho-ERK, #4377; anti-IκB, #9242; anti-phospho-IκB, #9241 anti-NF-κB p65, #3034; Danvers, MA, USA), Santa Cruz Biotechnology (GAPDH, sc-32233; TLR-4, sc-10741; Dallas, TX, USA), and Invitrogen (Lamin B1, #33-2000; Carlsbad, CA, USA).

### 4.5. Immunofluorescence Analysis

The HaCaT cells were seeded at a density of 1.5 × 105 cells/well in a two-chamber slide (Eppendorf, Hamburg, Germany). After 24 h, melittin and PgLPS were applied. The treated cells were washed with phosphate-buffered saline (PBS) and fixed with 4% paraformaldehyde for 20 min at room temperature. The fixed cells were treated with 0.1% Triton X-100 in PBS for 2 min to permeabilize. Following permeabilization, the cells were blocked in PBS containing 5% BSA at room temperature for 1 h. After blocking, the cells were incubated with a diluted primary antibody overnight at 4°C, then with a secondary antibody for 4 h at room temperature. The nuclei were stained with Hoechst 33342 solution (Thermor Fisher Scientific, Waltham, MA, USA) for 20 min. The slides were mounted using a fluorescence mounting medium (Dako, Santa Clara, CA, USA). The antibodies used were anti-β-actin (A5316; Sigma, St. Louis, MO, USA), anti-NF-κB p65 (#9139, Cell Signaling Technology, Danvers, MA, USA), anti-mouse, and anti-rabbit IgG Alexa Fluor 488 and 647 (Thermo Fisher Scientific, Waltham, MA, USA).

### 4.6. Statistical Analysis

All results were presented as mean ± standard error of the mean (SEM). Student’s *t*-test was used to analyze the significance of separate experiments. Differences among values were considered statistically significant when *p* < 0.05.
